# Axis I diagnosis profile according to the Diagnostic Criteria for Temporomandibular Disorders (DC/TMD): comparison between hospital-based orofacial pain clinic and dental academic-based orofacial pain clinic

**DOI:** 10.22514/jofph.2024.040

**Published:** 2024-12-12

**Authors:** Shoshana Reiter, Samah Jazmawi, Ephraim Winocur, Orit Winocur Arias, Lazar Kats, Yifat Manor

**Affiliations:** ^1^Department of Oral Pathology, Oral Medicine and Maxillofacial Imaging, The Maurice and Gabriela Goldschleger School of Dental Medicine, the Faculty of Medical and Health Sciences, Tel Aviv University, 6934228 Tel Aviv, Israel; ^2^The Maurice and Gabriela Goldschleger School of Dental Medicine, the Faculty of Medical and Health Sciences, Tel Aviv University, 6934228 Tel Aviv, Israel; ^3^Department of Oral Rehabilitation, The Maurice and Gabriela Goldschleger School of Dental Medicine, the Faculty of Medical and Health Sciences, Tel Aviv University, 6934228 Tel Aviv, Israel; ^4^Department Oral and Maxillofacial Surgery, The Maurice and Gabriela Goldschleger School of Dental Medicine, the Faculty of Medical and Health Sciences, Tel Aviv University, 6934228 Tel Aviv, Israel; ^5^Unit of Oral and Maxillofacial Surgery, Shamir Medical Center, 7033001 Beer Yaacov, Israel

**Keywords:** Diagnostic criteria for temporomandibular disorders (DC/TMD), Axis I, ICOP

## Abstract

Temporomandibular disorder (TMD) is considered a complex disorder that follows the biopsychosocial model. The current study aimed to explore the effect of clinic location and referring physicians on the distribution of Axis I diagnoses according to the Diagnostic Criteria for TMD (DC/TMD). Eighty-eight patients from a dental school Orofacial Pain Clinic (DentalOFP) and 104 patients from a hospital Orofacial Pain Clinic (HospitalOFP) were examined by the same dentist who was certified as a DC/TMD examiner and compared. Significant differences between the two clinics were noted, including age (*p* = 0.002), gender (*p* = 0.019), symptom duration (*p *< 0.001), and referring physician’s profile (*p* < 0.001). While 55.7% of referring physicians were dentists in the DentalOFP clinic, only 13.5% of referring physicians were dentists in the HospitalOFP clinic. DentalOFP clinic presented with characteristics of a tertiary clinic, as to female: male ratio and longer symptom duration. Significant differences were found as to intra-articular disorders (IAD) (*p* = 0.019), degenerative joint disorder (DJD) (*p* = 0.041), and subluxation (*p *= 0.015). There were no significant differences as to local myalgia (*p *= 0.128), myofascial pain with referral (*p* = 0.389), and arthralgia (*p *= 0.096). Multiple parameters, such as age, gender, symptom duration, primary *vs.* tertiary clinic, clinic location, and referring physicians may affect the overall DC/TMD Axis I profile. This study supports abandoning the term TMD. It is suggested to assess each Axis I diagnosis separately, and for each Axis I diagnosis, to follow the International Classification of Orofacial Pain (ICOP), as to primary *vs.* secondary etiologies, and acute *vs.* chronic conditions, to provide appropriate treatment.

## 1. Introduction

Temporomandibular disorder (TMD) is currently considered a complex disorder with multiple etiologies that follow the biopsychosocial model [1]. TMD is defined as “a group of musculoskeletal and neuromuscular conditions that involve the temporomandibular joints (TMJs), the masticatory muscles, all associated structures of mastication, and associated tissues” [2].

The Research Diagnostic Criteria for TMD (RDC/TMD) [3] was established to overcome multiple classifications of TMD [4, 5] that posed a major obstacle for research by creating uniform diagnostic criteria. The RDC/TMD was accepted worldwide for more than 2 decades as the preferred diagnostic criteria for research purposes. The DC/TMD [6], the revised version of the RDC/TMD, which was published in 2014, improved the sensitivity and specificity of diagnostic criteria, in part by refining the criteria for the identification of musculoskeletal pain-related disorders. In addition, diagnoses were divided into painful and non-painful TMD, although multiple diagnoses for the same patients were still permitted [7]. Nevertheless, criticism was raised by leading researchers [8, 9, 10] as to grouping pain-related diagnoses and joint-related diagnoses under the umbrella term “TMD” and as to the use of the term “TMD”. Laskin proposed to eliminate the term “TMD” and to evaluate myogenous and arthrogenous findings as independent musculoskeletal conditions [8]. Huff & Benoliel noted that the term TMD should not be viewed as an adequate diagnosis, but merely as a classification and that there are over 30 diagnoses under the term “TMD” [10]. This notion had been implemented in the International Classification of Orofacial Pain (ICOP), 1st edition [11] that stated that the term TMD is maintained only to align with the DC/TMD protocol. In addition, the ICOP adopted a clear division between muscle-related diagnoses (Myofascial orofacial pain) and painful TMJ-related diagnoses (Temporomandibular joint (TMD) pain), while differentiating between primary and secondary pain etiologies for each diagnosis, and also addressed chronicity status for each diagnosis.

Indeed, one of the main obstacles that restrict and hampers our understanding of TMD-related disorders is derived from viewing TMD as a single diagnostic entity and combining painful and non-painful diagnoses in the same patient. This approach may lead to falsely relating these diagnoses to each other as cause and effect, while dual diagnoses may represent comorbidity or incidental findings only [12]. Another important issue that is not addressed by the DC/TMD relates to pain mechanisms. As stated by Svensson [13]: “the specific criteria rely on clustering of specific symptoms and clinical signs without addressing putative underlying pain mechanisms”. Svensson emphasizes a specific painful TMD diagnosis may reflect a heterogenous pain mechanism which clinically may appear very similar [13]. Thus, current diagnostic criteria, although improved, are still lacking important information as to etiology, chronicity and underlying pain mechanisms even when addressing specific pain diagnosis listed as painful TMD. Each diagnosis listed in the DC/TMD can represent a primary or secondary condition, nociceptive pain or inflammatory pain. It can represent a neuropathic pain or even a nociplastic pain condition [14]. Moreover, in a broader view, TMD-related conditions may coexist with other chronic pain conditions such as fibromyalgia, irritable bowel syndrome, myalgic encephalomyelitis/chronic fatigue syndrome, vulvodynia, interstitial cystitis/painful bladder syndrome, chronic tension-type headache, migraine headache, endometriosis and chronic lower back pain. Associated with these patients are elevated levels of Axis II components, such as anxiety and depression. These conditions are referred to as chronic overlapping pain conditions (COPCs) [15]. This important information is not addressed by the DC/TMD. Failure to acknowledge COPSs and address TMD only might compromise treatment outcomes.

Another issue that supports abandoning the term TMD is the realization that many variables may change the composition of TMD as a group of diagnoses: age, gender, clinic location (family physician *vs.* dental academic center or hospital-based clinic), and clinic type: primary clinic, in which most cases have a good prognosis, *vs.* tertiary clinic, which is composed of a higher proportion of chronic primary pain patients [16], who may require a variety of treatment strategies to address the psychosocial components associated with their chronic pain condition [17]. These variables may hamper our ability to compare between studies, even when unified diagnostic criteria, such as the DC/TMD is used.

Additional factors that may affect the different diagnoses profile of TMD-related diagnoses that haven’t been addressed so far to the best of our knowledge are clinic location and the referring physician profile. It is logical to assume that dental academic centers that rely on dentists as the major referring source will show a higher percentage of mechanical TMJ findings, such as joint noises and limited mouth opening, while referred otalgia will be one of the major etiologies of referral from otolaryngologists or family physicians once primary otalgia was ruled out.

The purpose of this study, therefore, was to explore demographic and Axis I diagnoses differences between two orofacial pain clinics, each located in a different setting: the first, located in a dental school in which the majority of referring physicians are dentists, while the second clinic is located in a hospital-based Orofacial pain clinic within Oral surgery department, in which the majority of referring Physicians are not dentists.

## 2. Materials and methods

This retrospective study included two Orofacial pain clinics located in two separate cities: A university dental school-based Orofacial pain clinic (DentalOFP), and a hospital-based Orofacial pain clinic, which is a subdivision of an Oral surgery department (HospitalOFP). All the patients in both clinics were examined by the same senior staff member (EW) who worked in both clinics and is certified in the DC/TMD Training and Calibration Course at the Department of Orofacial Pain and Jaw Function at the Faculty of Odontology at Malmö, Sweden. Each patient who was seen in both clinics was diagnosed according to the Hebrew version [18] of the DC/TMD [6]. Patients’ medical records were retrospectively analyzed and compared.

The nonpainful Axis I diagnoses included intra-articular disorders (IAD) (disc displacement with reduction, disc displacement with reduction with intermittent locking, disc displacement without reduction with limited opening, and disc displacement without reduction without limited opening), degenerative joint disease (DJD) and subluxation. Painful Axis I diagnoses included in the analysis included arthralgia, local myalgia and myofascial pain with referral.

Overall, 164 patients were examined in the HospitalOFP clinic by EW during 2019–2020 (pre-COVID-19 pandemic). Excluded from the study were 13 patients younger than 18 years, 28 did not meet the criteria to receive an Axis I diagnosis of TMD according to the DC/TMD specifications and were diagnosed as having other orofacial pain conditions. Nine patients were excluded due to receiving a diagnosis of bruxism only. The data of 10 patients was missing information necessary for analysis, such as symptom duration, and therefore were excluded from the study. The final study population of the HospitalOFP clinic included 104 patients (Fig. 1).

**Fig. 1.** Flowchart for HospitalOFP and DentalOFP clinics. DentalOFP: Dental school orofacial pain clinic; HospitalOFP: Hospital orofacial pain clinic. All 172 patients were examined by Prof. EW. *Excluded: (1) 13 patients younger than 18 years. (2) 28 did not meet the criteria to receive an Axis I diagnosis of TMD according to the DC/TMD specifications and were diagnosed as having other orofacial pain conditions. (3) 9 patients were excluded due to receiving a diagnosis of bruxism only. (4) 10 patients—missing information necessary for analysis such as symptoms duration, and therefore were excluded from the study. **Excluded: (1) 11 patients younger than 18 years. (2) 35 did not meet the criteria to receive an Axis I diagnosis of TMD according to the DC/TMD specifications and were diagnosed as having other orofacial pain conditions. (3) 15 patients were excluded due to receiving a diagnosis of bruxism only, and one patient was excluded due to obstructive sleep apnea as the only diagnosis. (4) 22 patients—missing information necessary for analysis, such as symptom duration, and therefore were excluded from the study.

Overall, 172 patients were examined in the DentalOFP clinic by WE during 2015–2018 (pre-COVID-19 pandemic). Excluded from the study were 11 patients younger than 18 years, 35 did not meet the criteria to receive an Axis I diagnosis of TMD according to the DC/TMD specifications and were diagnosed as having other orofacial pain conditions such as neuropathic pain, systemic diseases such as rheumatoid arthritis, fibromyalgia, migraine, burning mouth syndrome, dental diseases and others). Fifteen patients were excluded due to receiving a diagnosis of bruxism only, one patient was excluded due to obstructive sleep apnea as the chief complaint, with no TMD signs and symptoms. The data of 22 patients was missing information necessary for analysis such as symptoms duration, and therefore were excluded from the study. The final study population of the DentalOFP clinic included 88 patients (Fig. 1).

Categorical variables were summarized as frequency and percentage. Continuous variables were evaluated for normal distribution using histogram and Kolmogorov-Smirnov Test and reported as median and interquartile range. The Chi-Square Test and Fisher’s Exact Test were used to compare categorical variables between the two clinics and the Mann-Whitney test was used to assess differences in continuous variables. Multivariable Logistic Regression was used to study the association between the clinics and the diagnosis while controlling age, gender, symptoms duration and referring physician. IBM SPSS Statistics for Windows, Version 28.0. Armonk, NY, USA: IBM Corp. was used for all statistical analyses. A *p*-value < 0.05 was considered statistically significant.

## 3. Results

### 3.1 Stage 1—data summarization

Fig. 2 summarizes demographic information for both clinics: In the DentalOFP clinic male: female ratio was 1:4.88, mean age (of 39.25 ± 15.98), while in the HospitalOFP clinic male: female ratio was 1:2.15, and the mean age (47.89 ± 18.49). Significant differences between the two clinics were shown as to age, gender, symptom duration, and referring physicians; Overall, DentalOFP patients were significantly younger (*p *= 0.002), and 87.5% reported symptom duration which was over 3 months, compared to 69.2% of HospitalOFP patients (*p* < 0.001). 55.7% of referring sources in the DentalOFP clinic were dentists, while in the HospitalOFP clinic, only 13.5% were referred by dentists. While 84.6% of referring physicians in the HospitalOFP clinic were family physicians/specialized physicians (such as ear, nose and throat specialists (ENT) and neurologists), only 10.2% of the patients in the DentalOFP clinic were referred by General/Specialized physicians.

**Fig. 2.** Comparison between DentalOFP and HospitalOFP clinics: gender, age, symptom duration, and referring physician. Gender: *p* = 0.019; Age: *p* = 0.002; Symptom duration: *p* < 0.001; Referring physician: *p* < 0.001. DentalOFP: Dental school orofacial pain clinic; HospitalOFP: Hospital orofacial pain clinic.

As to Axis I diagnoses, Fig. 3 presents percentages of all Axis I diagnoses. significant differences were found for all non-painful Axis I diagnoses individually, including, IAD (*p *= 0.019), DJD (*p *= 0.041), and subluxation (*p *= 0.015). 37.5% of the patients in DentalOFP were diagnosed with IAD compared to 22.1% in HospitalOFP (*p *= 0.019), 26.9% of the patients from HospitalOFP received a diagnosis of DJD compared to 14.8% in DentalOFP (*p *= 0.041) and 13.5% of HospitalOFP’s patients were diagnosed with subluxation while only 3.4% were in DentalOFP (*p* = 0.015). However, when analyzing painful and non-painful diagnoses as two separate groups: In the group of patients receiving only non-painful diagnoses (IAD, DJD, subluxation), there were no significant differences (*p *= 0.058), while in the group of patients receiving painful diagnoses (local myalgia, myofascial pain with referral, and arthralgia), significant differences were found (*p *= 0.040).

**Fig. 3.** Axis I diagnoses: comparison between DentalOFP clinic and HospitalOFP clinic. IAD: Intra-articular disorders; DJD: Degenerative joint disease; HospitalOFP: Hospital orofacial pain clinic; DentalOFP: Dental school orofacial pain clinic.

### 3.2 Stage 2—multivariable logistic regression while controlling for gender and age

At this stage, a Multivariable Logistic Regression analysis was performed while controlling for gender and age. The results of the multivariable logistic regression are presented in Table 1. The odds for diagnosis of local myalgia in the DentalOFP clinic were 1.47 times higher than in the HospitalOFP clinic (*p* = 0.205). The odds for diagnosis of myofascial pain with referral at the DentalOFP clinic was 1.18 times higher compared to HospitalOFP (*p* = 0.627). As to arthralgia, the odds in the DentalOFP clinic were 2.58 times higher than at the HospitalOFP clinic (*p* = 0.018). For diagnosis of DJD, the odds in the HospitalOFP clinic were 1.63 times higher than at the DentalOFP clinic (*p* = 0.215). The odds for IAD diagnosis in the DentalOFP clinic were 1.48 times higher than in the HospitalOFP clinic (*p* = 0.268). However, the odds for subluxation in the HospitalOFP clinic were 5.31 times higher than in the DentalOFP clinic (*p* = 0.015).

**Table 1.** Multivariable logistic regression while controlling for gender and age (HospitalOFP).

Axis I diagnoses (DC/TMD) [6]	OR (95% CI)	*p*
Local myalgia	0.678, CI (0.372–1.236)	0.205
Myofascial pain with referral	0.846, CI (0.431–1.661)	0.627
Arthralgia	0.388, CI (0.177–0.850)	0.018
DJD	1.626, CI (0.755–3.505)	0.215
IAD	0.676, CI (0.338–1.351)	0.268
Subluxation	5.310, CI (1.380–20.425)	0.015
Painful disorders: (local myalgia, myofascial pain with referral, arthralgia)	0.412, CI (0.172–0.984)	0.046
Only non-painful disorders (has DJD and/or IAD and/or subluxation, and doesn’t have painful disorders)	2.271, CI (0.946–5.454)	0.067

*IAD: Intra-articular disorders; DJD: Degenerative joint disease; HospitalOFP: Hospital orofacial pain clinic; DC/TMD: Diagnostic Criteria for Temporomandibular Disorders; OR: Odds Ratio; CI: Confidence Interval.*

As to Painful Axis I Diagnoses as a group (local myalgia, myofascial pain with referral, and arthralgia), the odds in the DentalOFP clinic were 2.43 higher than in the HospitalOFP clinic (*p* = 0.046). On the other hand, the odds for non-painful Axis I Diagnoses (DJD, IAD and Subluxation) in the HospitalOFP clinic were 2.27 higher than in the DentalOFP clinic (*p* = 0.067).

### 3.3 Stage 3—multivariable logistic regression while controlling for gender, age and symptom duration

At this stage, a Multivariable Logistic Regression analysis was performed while controlling for gender, age and symptom duration. Results are presented in Table 2.

**Table 2.** Multivariable logistic regression while controlling for gender, age and symptom duration (HospitalOFP).

Axis I diagnoses (DC/TMD) [6]	OR (95% CI)	*p*
Local myalgia	0.651, CI (0.348–1.219)	0.180
Myofascial pain with referral	0.907, CI (0.448–1.835)	0.786
Arthralgia	0.381, CI (0.166–0.877)	0.023
DJD	1.743, CI (0.779–3.899)	0.176
IAD	0.876, CI (0.416–1.843)	0.727
Subluxation	6.809, CI (1.683–27.544)	0.007
Painful disorders: (local myalgia, myofascial pain with referral, arthralgia)	0.415, CI (0.165–1.043)	0.061
Only non-painful disorders (has DJD and/or IAD and/or subluxation, and doesn’t have painful disorders)	2.178, CI (0.858–5.531)	0.102

*IAD: Intra-articular disorders; DJD: Degenerative joint disease; HospitalOFP: Hospital orofacial pain clinic; DC/TMD: Diagnostic Criteria for Temporomandibular Disorders; OR: Odds Ratio; CI: Confidence Interval.*

The odds for diagnosis of local myalgia in the DentalOFP clinic were 1.54 times higher than in the HospitalOFP clinic (*p* = 0.180). The odds for diagnosis of myofascial Pain with referral in the DentalOFP clinic was 1.10 times higher than in the HospitalOFP clinic (*p* = 0.786). For arthralgia, the odds in the DentalOFP clinic were 2.62 times higher than in the HospitalOFP clinic (*p* = 0.023). For diagnosis of DJD, the odds in the HospitalOFP clinic were 1.74 times higher than in the DentalOFP clinic (*p* = 0.176). The odds for IAD diagnosis in the DentalOFP clinic were 1.14 times higher than in the HospitalOFP clinic (*p* = 0.727). However, the odds for subluxation in the HospitalOFP clinic were 6.81 times higher than in the DentalOFP clinic (*p* = 0.007).

As to Painful Axis I Diagnoses as a group (local myalgia, myofascial pain with referral, and arthralgia), the odds in the DentalOFP clinic were 2.41 times higher than in the HospitalOFP clinic (*p* = 0.061). On the other hand, the odds for only non-painful Axis I Diagnoses (DJD, IAD and Subluxation) in the HospitalOFP clinic were 2.18 times higher than in the DentalOFP clinic (*p* = 0.102).

### 3.4 Stage 4—multivariable logistic regression while controlling for gender, age, symptoms duration and referring physician

At this stage, a Multivariable Logistic Regression analysis was performed while controlling gender, age, symptom duration and referring Physician. Results are presented in Table 3.

**Table 3.** Multivariable logistic regression while controlling for gender, age, symptom duration and referring physician (HospitalOFP).

Axis I diagnoses (DC/TMD) [6]	OR (95% CI)	*p*
Local myalgia	0.284, CI (0.104–0.777)	0.014
Myofascial pain with referral	1.343, CI (0.490–3.677)	0.567
Arthralgia	0.290, CI (0.088–0.955)	0.042
DJD	2.330, CI (0.765–7.100)	0.137
IAD	1.004, CI (0.336–2.995)	0.995
Subluxation	9.154, CI (1.152–72.733)	0.036
Painful disorders: (local myalgia, myofascial pain with referral, arthralgia)	0.179, CI (0.047–0.683)	0.012
Only non-painful disorders (has DJD and/or IAD and/or subluxation, and doesn’t have painful disorders)	5.413, CI (1.395–21.003)	0.015

*IAD: Intra-articular disorders; DJD: Degenerative joint disease; HospitalOFP: Hospital orofacial pain clinic; DC/TMD: Diagnostic Criteria for Temporomandibular Disorders; OR: Odds Ratio; CI: Confidence Interval.*

The odds for diagnosis of local myalgia in DentalOFP clinic were 3.52 times higher than in the HospitalOFP clinic (*p* = 0.014). The odds for diagnosis of myofascial pain with referral in the HospitalOFP clinic was 1.34 times higher than in the DentalOFP clinic (*p* = 0.567). For diagnosis of arthralgia, the odds in DentalOFP clinic were 3.45 times higher than in the HospitalOFP clinic (*p* = 0.042). For diagnosis of DJD, the odds in the HospitalOFP clinic were 2.33 times higher than in the DentalOFP clinic (*p* = 0.137). For IAD, the odds were approximately equal in DentalOFP (Odds Ratio (OR) = 0.995) and in HospitalOFP (OR = 1.004, *p* = 0.995) However, the odds for diagnosis of subluxation in HospitalOFP clinic were 9.15 times higher than in the DentalOFP clinic (*p* = 0.036).

As to Painful Axis I Diagnoses as a group (local myalgia, myofascial pain with referral, and arthralgia), the odds in the DentalOFP clinic were 5.59 times higher than in the HospitalOFP clinic (*p* = 0.012). The odds for non-painful Axis I Diagnoses (DJD, IAD and Subluxation) in the HospitalOFP clinic were 5.41 times higher than in the DentalOFP clinic (*p* = 0.015).

## 4. Discussion

In the current study two orofacial pain clinics, each from a different setting, were compared. Patients in both clinics were examined by the same physician and received diagnoses of Axis I according to the DC/TMD. Results showed that both clinics were significantly different from each other by many parameters, including average age, female: male ratio, symptom duration, referring physicians, and all non-painful diagnoses; As to gender, gender plays an important role in TMD [19]. Studies have repeatedly shown that females have a higher risk for TMDs in general than males [20]. The age and gender profile of the DentalOFP group was more typical to what is known so far [19]. On the other hand, it is noteworthy that most studies on TMD patients using the RDC/TMD or the DC/TMD were conducted in academic centers and not in hospital-based orofacial pain clinics [21]. The profile of Axis I diagnoses in the DentalOFP clinic shown in the current study was similar to these studies, as opposed to the profile of the HospitalOFP clinic. As was shown, the proportion of women increases from community-based study to primary and tertiary clinical settings, with women comprising approximately 80% or more of patient populations in tertiary academic clinics [22]. Considering this with longer pain duration in the DentalOFP clinic, may point to the characteristics profile of tertiary clinic in the DentalOFP clinic compared to the HospitalOFP clinic [23]. The average younger age compared to the HospitalOFP clinic could explain the higher prevalence of IAD diagnoses found in the DentalOFP clinic since the average age tends to be younger when it comes to IAD [24], while the higher average age in the HospitalOFP clinic could explain the higher prevalence of DJD since DJD are more prevalent in adults/elderly [25]. Indeed, after controlling for age and gender, these significant differences between IAD and DJD were eliminated. As to diagnosis of subluxation, one might argue that the higher prevalence of diagnoses of subluxation in the HospitalOFP clinic may reflect referral of cases such as recurrent dislocation of the TMJ who did not respond to conservative treatment in primary/secondary clinics and therefore were referred to the HospitalOFP clinic which is a subdivision of oral surgery department for considering surgery. The significantly higher odds of subluxation in the HospitalOFP which remained after controlling for age, gender, symptom duration and referring physicians, may support this theory. However, the finding of subluxation could also be an incidental finding unrelated to the patient’s chief complaint, or it might represent a functional/nociplastic disorder. Therefore, it should be noted that while the DC/TMD enables diagnosis of non-painful findings, such as IAD, DJD and subluxation, it cannot differentiate between merely incidental physical findings or findings that may be the etiologic source for secondary painful joint pain. In that respect assessing primary *vs.* secondary pain is essential for tailoring treatment. This aspect has been addressed by the ICOP [11], as mentioned in the introduction section. Thus, according to the DC/TMD, the finding of subluxation is recorded and considered a non-painful diagnosis, while according to the ICOP, subluxation could be grouped under the category of secondary TMJ pain: “Temporomandibular joint pain attributed to subluxation”. As to other etiologies for non-painful diagnoses according to the DC/TMD, there is still missing diagnostic criteria to address these and to differentiate between primary and secondary etiologies. For example, generalized hypermobility in a patient can explain a finding of subluxation, while another etiology for subluxation could be facial trauma.

Compared to non-painful diagnoses, there were no significant differences as to all painful diagnoses, including local myalgia, myofascial pain with referral, and arthralgia. After controlling for age and gender, the odds for diagnosis of arthralgia were significantly higher in the DentalOFP clinic. Further controlling for referring physician resulted in higher odds of a diagnosis of local myalgia. These findings do not support our hypothesis of a higher prevalence of arthralgia in clinics where the majority of referring physicians are not dentists (*i.e.*, HospitalOFP). Again, once understanding that diagnoses categorized under the umbrella of painful TMD according to the DC/TMD cannot differentiate between different etiologies of painful diagnoses such as arthralgia of the TMJ and myofascial pain with referral, as listed in the ICOP and different pain mechanisms, it is problematic to extract any conclusion using the DC/TMD when an attempt to compare between these two clinics is made, as was done in the current study, even though the same clinician examined all the patients, and used a unified diagnostic criteria (DC/TMD).

It is interesting to note, that controlling for age, gender, symptoms duration, and referring physicians did not affect the diagnosis of myofascial pain with referral which was the only diagnosis that remained nonsignificant when comparing between the two groups even after controlling for the above parameters. However, while using the DC/TMD information is lacking for each diagnosis regarding the definition of pain as acute *vs.* chronic primary or secondary, which is crucial information that may determine the treatment approach [26]. This important information for each diagnosis is lacking in the DC/TMD, while addressed by the ICOP which differentiates between acute and chronic myofascial orofacial pain conditions and further subdividing diagnosis according to chronicity and etiology into primary *vs.* secondary, and according to chronicity into acute *vs.* chronic (frequent/infrequent/highly frequent, and chronic persistent). Likewise, recognizing the type of pain, as suggested by Woolf *et al*. [27] as nociceptive pain, inflammatory pain, neuropathic pain or functional pain/nociplastic pain [28], while improved compared to the RDC/TMD, is still limited using the DC/TMD.

Another major contributor to differences between the two groups that was explored in the current study was the profile of referring physicians. It would be logical to assume that dentists will be more prone to refer patients with mechanical joint complaints, such as clicking and crepitation, while general physicians/ear nose, and throat specialists would tend to refer patients with referred otalgia complaints, once primary otalgia was ruled out. Therefore, the younger age profile of the DentalOFP patients could be the result of referral of patients with IAD by dentists, and not the cause. In the current study, however, controlling for referring physicians, had a significant effect on local myalgia only. Overall, non-painful diagnoses were more sensitive to age, and gender compared to painful diagnoses. These findings highlight the heterogenicity of non-painful TMJ-related diagnoses. Indeed, in a systematic review [5] that examined the prevalence of intra-articular related diagnoses (arthralgia, IAD, DJD and subluxation) among the general population, by analyzing and comparing studies that used the RDC/TMD to studies that used the DC/TMD, showed great variability in prevalence of intra-articular related diagnoses in both RDC/TMD and DC/TMD studies: In RDC/TMD studies the prevalence of Arthralgia diagnosis ranged between 5.7–17%, the prevalence of disc displacement with reduction ranged between 2.1–33%, and prevalence of osteoarthrosis ranged between 4.8–70%. This is compared to DC/TMD studies: arthralgia, 1.2–21.1%, disc displacement with reduction, 20.8–47.9%, DJD, 1.3–34.9%. Data in this meta-analysis was not available as to muscle-related disorders, unfortunately. It seems, that as to non-painful Axis I diagnoses, a great heterogeneity exists, even when using standardized diagnostic criteria. In the current study, these differences were shown even though the same certified dentist examined the patients in both clinics while using the same diagnostic criteria. This highlights the importance of parameters such as age, gender, clinic type and referring physicians in determining non-painful diagnosis profiles. This also highlights the need for establishing specific primary and secondary diagnostic criteria for different etiologies for non-painful TMJ findings in addition to what was offered for painful diagnoses by the ICOP.

In that sense, the current study supports abandoning the term TMD altogether by highlighting the problematic issues that may arise by using the DC/TMD as is, including assigning multiple diagnoses to the same patient without addressing etiology, chronicity and pain mechanisms.

As with any study, the current study has strengths and limitations: The current study aimed for a high standardization by using DC/TMD protocol and collecting data of patients who were examined by the same physician. This undoubtedly increased the reliability of the study as to diagnoses in both clinics. However, unfortunately, the data for Axis II parameters, including levels of depression, anxiety, nonspecific physical symptoms, pain levels, and disability, in the HospitalOFP clinic were not available due to hospital clinic limitations. Currently, TMD-related diagnoses are assessed according to the biopsychosocial model [29]. This is a major limitation of this study since Axis II parameters and pain intensity contribute to disability and chronicity and may explain in part symptom duration differences that were found between the two groups and the prevalence of painful diagnoses [30]. There is an intimate association between Axis II profile and painful diagnoses that was not analyzed in the current study. Future studies should strive to include Axis II findings in the analysis.

## 5. Conclusions

Multiple parameters affect the profile of an orofacial pain clinic, such as clinic type (primary *vs.* tertiary), clinic location, age, gender, symptom duration and referring physician’s profile. Orofacial pain clinicians in dental academic centers should be aware of skewed diagnoses of the Axis I profile due to these parameters. This study supports abandoning the term TMD, as pointed out by several researchers. It is suggested to assess each diagnosis according to the DC/TMD separately while avoiding division into painful and non-painful diagnoses. While allowing multiple diagnoses in the same patient, it is suggested to analyze each diagnosis separately, as suggested by the ICOP, including specific information as to primary or secondary etiologies, chronicity and pain mechanisms involved. It should be remembered that the same diagnosis according to the DC/TMD can have different etiologies, all presenting similar clinically. Future information as to pain mechanisms, chronicity status, Axis II profile, and the existence of other chronic overlapping pain conditions can further assist in tailoring the appropriate treatment.

## 6. Highlights

• Multiple parameters, such as age, gender, symptom duration, Clinic type (primary *vs.* tertiary), clinic location (hospital *vs.* academic center), and referring physicians’ profile may affect the overall Axis I diagnostic profile of TMD patients within the clinic.

• Orofacial pain clinicians in dental academic centers should be aware of skewed diagnoses of the Axis I profile due to these parameters.

• This study supports abandoning the term TMD. It is suggested to assess each diagnosis according to the DC/TMD separately while avoiding division into painful and non-painful diagnoses.

• For each DC/TMD Axis I diagnosis, it is suggested to follow the ICOP diagnostic criteria, as to primary and secondary etiologies, and acute *vs.* chronic conditions.
